# Supplementary material for: MetaRibo-Seq measures translation in microbiomes
Source: Nat Commun. 2020 Jun 29;11:3268. doi: 10.1038/s41467-020-17081-z (PMC7324362; doi:10.1038/s41467-020-17081-z)
Supplement: Supplementary file 10 — Supplementary Data 7 [file 41467_2020_17081_MOESM10_ESM.zip › File2/Confidence_High_Taxonomy/381474_out.krona.html]

Javascript must be enabled to view this page.

members
magnitude
magnitudeUnassigned
count
unassigned
taxon
rank

381474\_out

3

3
superkingdom
2

3
phylum
976

class
3
200643

171549
order
3

171552
3
family

genus
3
838

3

SRS023466\_contig\_number\_279SRS023604\_contig\_number\_377SRS142710\_contig\_number\_contig-100\_929.28743
species
1703337
